# Cost analyses of plasma p-tau217 versus p-tau217/Aβ42 ratio using two-step approach in the Japanese health care system

**DOI:** 10.1016/j.tjpad.2026.100572

**Published:** 2026-04-18

**Authors:** Masanori Kurihara, Ryoko Ihara, Kenichiro Sato, Atsushi Iwata

**Affiliations:** a Department of Neurology, Tokyo Metropolitan Institute for Geriatrics and Gerontology, 35-2, Sakaecho, Itabashi-ku, Tokyo 173-0015, Japan; b Integrated Research Initiative for Living Well with Dementia, Tokyo Metropolitan Institute for Geriatrics and Gerontology, 35-2, Sakaecho, Itabashi-ku, Tokyo 173-0015, Japan; c Healthy Aging Innovation Center, Tokyo Metropolitan Institute for Geriatrics and Gerontology, 35-2, Sakaecho, Itabashi-ku, Tokyo 173-0015, Japan; d Dementia Inclusion and Therapeutics, The University of Tokyo Hospital, 7-3-1, Hongo, Bunkyo-ku, Tokyo 113-8655, Japan

**Keywords:** Alzheimer’s disease, Blood biomarkers, P-tau217, Cost-effectiveness, Cerebrospinal fluid, Positron-emission tomography, anti-amyloid-beta

## Abstract

Plasma p-tau217 may offer a cost-saving effect in diagnosing Alzheimer’s disease. However, each healthcare system has different costs, and its impact on evaluating anti-amyloid β (Aβ) therapies in Japan remains unclear. We conducted cost analyses using a two-step approach with a recently released application, assuming that measuring two analytes (p-tau217/Aβ42) would reduce the intermediate zone to 7%, despite doubling the price. Plasma biomarker costs were simulated from 100 to 800 USD. Cost savings ranged 34–79% compared with positron electron tomography (PET) and -5.6%–74% compared with in-patient cerebrospinal fluid (CSF) Aβ42/40 when 14.7% were in the intermediate zone. Savings were comparably high by measuring two analytes at 100 or 200 USD per analyte and gradually differed (one analyte better savings than two) as the cost per analyte increased. Both plasma p-tau217 and p-tau217/Aβ42 showed substantial cost-saving effects, with comparably high savings at lower costs (100, 200 USD) per analyte.

## 1 Introduction

Anti-amyloid β (Aβ) therapies (AAT), lecanemab and donanemab, have demonstrated clinical efficacy in patients with early Alzheimer’s disease (AD) [1,2]. Japan was the second country to approve these treatments, and the costs of amyloid PET or cerebrospinal fluid (CSF) biomarkers are now covered by the national health care system to confirm brain amyloid pathology necessary for the eligibility for AAT [3,4]. Blood-based biomarkers (BBMs), including plasma p-tau217, have recently shown excellent diagnostic accuracy for AD, particularly using the two-step approach [[5], [6], [7], [8], [9]]. For clinical implementation, recent recommendations require diagnostic accuracy equivalent to approved CSF biomarkers, that is at least 90% sensitivity and 90% specificity for confirmatory testing in specialized care settings [7,10,11]. In the two-step approach, two cutoffs are set to ensure this predetermined high sensitivity and specificity in the high and low groups, respectively, with only individuals in the intermediate zone requiring confirmatory tests (either PET or CSF) [5]. Plasma p-tau217/Aβ42 ratio offers the added advantage of reducing the number of individuals in the intermediate zone who need confirmatory tests [9], and the measurement of p-tau217/Aβ42 by the Lumipulse assay has been approved by the Food and Drug Administration (FDA) in the United States (US) to identify patients with amyloid pathology associated with AD [12].

The introduction of BBMs reduces the burden on patients and health care professionals and may even offer cost-saving benefits. Previous simulation studies have shown that BBMs are cost-effective in recruitment for secondary prevention clinical trials [13,14] or in real-world settings in the US [9,15]. However, given that each country has a different health care system, the cost-effectiveness of BBMs in real-world settings in Japan remains undetermined.

Therefore, we conducted cost analyses of plasma p-tau217 and p-tau217/Aβ42 ratio using two-step approach in the evaluation of AAT within the Japanese health care system.

## 2 Methods

We used the recently released app (https://bbrc-lab.shinyapps.io/Cost-effectiveness_analysis_plasma_p-Tau217/) [9] to estimate the cost-saving effect of BBMs when used as confirmatory testing in Japan. Another scenario would be the local authority to approve BBMs only as a triage test. In Japan, nearly all patients visiting hospitals/clinics use the national health insurance, and the cost for each test/procedure is determined at a fixed price (irrespective of the institution or region) by the Central Social Insurance Medical Council [16]. Based on data in our hospital, the cost mainly used in this study for amyloid PET (delivery flutemetamol) was 1550 USD, and for CSF Aβ42/40, it was 880 USD, including one-night stay for lumbar puncture (when USD 1 = JPY 147). Only delivery tracers were used in our hospital in the setting of evaluation of AAT where timely on demand testing is necessary. Lumbar punctures were performed in-patient settings, which is not uncommon in Japan [17]. These costs may vary in different situations, and other estimated costs, such as PET using synthesized tracers (also fixed price in Japan), in-patient CSF in hospitals with minimal surcharges, or out-patient CSF (fixed price) (Supplementary Table 1), were used in sensitivity analyses.

Considering that BBMs have additional benefits beside costs, the primary aim of this study was to identify the cost range with additional positive cost-saving effect. The cost of plasma p-tau217 used in a previous study evaluating cost-saving effects in the United States was 250 USD [9], and could be lower in Japan. Therefore, the cost of BBM was simulated from 100 USD and increased until the cost-saving effect became negative against either PET or CSF, in the main analysis. Since the cost-saving was already negative at 100 USD against lowest price estimates for CSF, the cost of BBM was simulated from 30 USD, which was the lowest price that could be selected in the application.

Pretest probability should be high in specialized memory-care evaluating eligibility for AAT. Based on the recently published Alzheimer’s Association guideline in specialized care settings [11], only estimates from the two-step approach ensuring high sensitivity (95%) and high specificity (95%) are reported in this manuscript evaluating as a confirmatory test. The cost-saving effects against PET or CSF were calculated using two different intermediate zone percentages, 14.7% and 7% [9]. Sensitivity analyses were performed based on 1) different costs for PET and CSF (Supplementary Table 1) and 2) different intermediate zone percentages recently reported in the Japanese [15% for p-tau217, 3% for p-tau217/Aβ42](18) and Chinese [13% for p-tau217, 10.7% for p-tau217/Aβ42] [19] populations.

## 3 Results

**Fig. 1:**
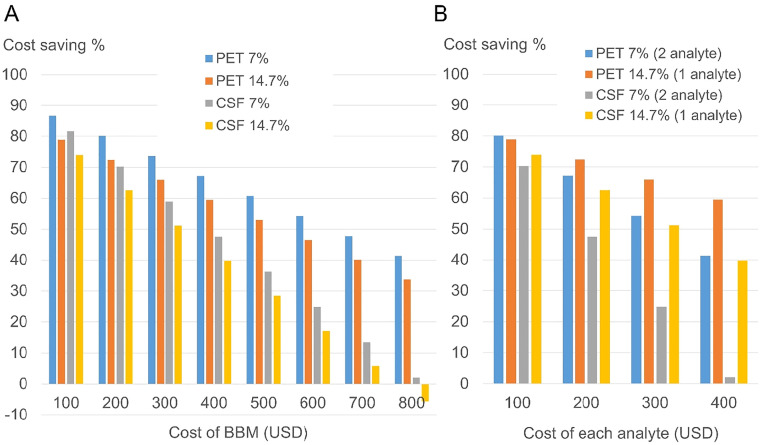
Cost-effectiveness analyses of blood-based biomarkers (BBM) against PET or CSF. (A) Cost savings when the proportion of individuals in the intermediate zone (those requiring confirmatory testing) was set at either 14.7% or reduced to 7% using the p-tau217/Aβ42 ratio. (B) Comparison of cost savings between measuring one analyte (p-tau217) and two analytes (p-tau217 and Aβ42) at varying costs per analyte.

The cost-saving remained positive at 40–79% against PET and 6%–74% against in-patient CSF in the BBM cost range of 100 to 700 USD, when the default setting of 14.7% was applied for the intermediate zone (Fig. 1A). When 7% was applied for the intermediate zone in reference to a recent report using p-tau217/Aβ42 in secondary clinics [9], the cost-saving was higher at 48–87% against PET and 14–82% against CSF (Fig. 1A). In sensitivity analyses, the cost-saving effect remained positive until 700 USD against PET, until 300 USD against in-patient CSF, and until 90–100 USD against out-patient CSF (Supplementary Table 2).

Based on the assumption that the cost of measuring two analytes (p-tau217/Aβ42) is required to reduce the percentage in the intermediate zone from 14.7% to 7% [9] despite doubling the price, the cost-saving effect was compared between measuring 1 versus 2 analytes. The cost-saving effects were comparably high at 70–80% at 100 USD per analyte, 48–72% at 200 USD per analyte, and gradually differed as the cost per analyte increased (Fig. 1B). In sensitivity analyses using different intermediate zone percentages, the cost-saving effect of 2 analytes remained comparable against PET or in-patient CSF at lower cost per analyte (100 USD), but not against out-patient CSF (Supplementary Table 3 and 4).

## 4 Discussion

In this study, we demonstrated that both plasma p-tau217 and p-tau217/Aβ42 have a large cost-saving effect as a confirmatory test in the Japanese health care system. At lower costs (100, 200 USD) per analyte, the cost-saving effect was comparably high using p-tau217/Aβ42, even after considering that measuring two analytes may double the cost to reduce the intermediate zone.

Previous estimates based on different costs showed that the cost-saving effect of plasma p-tau217 is approximately 81.1% against PET and 60.3% against CSF in the US health care system [9]. Based on the same assumption used in this study, which involves measuring two analytes (p-tau217 and Aβ42) to decrease the intermediate zone while doubling the price, the cost-saving effect of the FDA-approved plasma p-tau217/Aβ42 was approximately 85% against PET and 43% against CSF (Table 1). Our main estimates in this study showed similarly high cost-saving effects at the price of 100 or 200 USD per analyte in the Japanese health care system (Table 1). Given that reducing the intermediate zone also benefits timely diagnosis and reduces the burden on both patients and health care professionals, these cost-saving estimates may help guide the decision between plasma p-tau217 and p-tau217/Aβ42 and inform the pricing of these biomarkers in the Japanese health care system.

**Table 1:** Comparison of amyloid PET, CSF, plasma p-tau217, and p-tau217/Aβ42 costs and cost-saving estimates in the United States and Japan under different intermediate zone assumptions.

	US estimates	cost saving		Our estimates in Japan	cost saving	
		Vs PET	Vs CSF		Vs PET	Vs CSF
Amyloid PET	6000 USD [7]			1550 USD		
CSF	1000 USD [7]			880 USD		
Plasma p-tau217 intermediate zone 14.7%	250 USD [7]	81%	60%	100 USD	79%	74%
				200 USD	72%	63%
Plasma p-tau217/Aβ42 intermediate zone 7%	500 USD	85%	43%	200 USD	80%	70%
				400 USD	67%	48%

PET, positron electron tomography; CSF, cerebrospinal fluid.

Although confirmatory diagnosis using BBMs remains a matter of debate, recent reports have highlighted that some BBMs are non-inferior or may even be superior to currently used confirmatory CSF tests [6,20,21]. Based on these reports, recent criteria and guidelines propose that “accurate” or “high sensitivity and high specificity” BBMs (at least 90% sensitivity and 90% specificity) may be used as confirmatory tests [7,10,11]. While most BBMs have not consistently met these criteria using single cutoffs [11], some BBMs including p-tau217 [6,9] and p-tau217/Aβ42 [9] have met these criteria using predetermined two cutoffs consistently in different cohorts, and the Lumipulse p-tau217/Aβ42 was recently approved by the US-FDA [12]. Although the decision remains to be determined by each local regulatory authority, the use of these “accurate” BBMs in confirmatory testing for AAT is desired not only for reducing costs but also for better patient care.

During this study, we noticed significant differences in the cost of PET and CSF from previous estimates in other countries [9,[22], [23], [24], [25], [26], [27]]. In the current cost analyses of BBMs, the difference in the minimal cost of CSF, in particular, had a large impact on the cost of BBMs associated with cost-saving effects. Further studies using region-specific costs for PET and CSF in other countries would be important to understand the cost-saving effect of BBMs [9].

There are several limitations to this study. First, although the intermediate zone percentages were from recent clinical populations [9,18,19], the percentages could be slightly different in the real-world population assessed for AATs. Using data from the target population is crucial for better estimates. Second, although health care costs are fixed for a relatively long time in Japan (usually revised every two years) [16], the costs of PET and CSF may change in the future, requiring reanalysis. Third, this study focused on confirmatory testing using the two-step approach in the context of evaluation for AAT at secondary specialty clinics. Cost-effectiveness analyses in the scenario of prescreening/triaging, including dynamic simulations [28], may also be important. In addition, measurement of CSF p-tau181 is also covered for differentiating the cause of dementia in Japan, and BBMs may also have a cost-saving effect in this context. However, caution is needed for these estimations since the number of patients receiving biomarker-based diagnoses could dramatically increase by the introduction of BBMs in these situations.

## 5 Conclusion

Both plasma p-tau217 and p-tau217/Aβ42 were cost-effective in the context of confirmatory testing for AAT using two step approach in the Japanese health care system. Savings were comparably high using two analytes to reduce intermediate zone, at lower costs (100, 200 USD) per analyte. Further cost analyses in other countries and situations would be essential for the global implementation of BBMs for AD.

## Ethical statement

All human data used in this study were from open datasets. All procedures were performed in compliance with relevant laws and institutional guidelines, and ethical approval was waived.

## Data Availability Statement

All data supporting the findings of this study are included in the manuscript.

## Declaration of generative AI and AI-assisted technologies in the manuscript preparation process

AI was not used to generate any parts of this manuscript.

## Funding

This study was supported by the Integrated Research Initiative for Living Well with Dementia of the Tokyo Metropolitan Institute for Geriatrics and Gerontology and by the Davos Alzheimer’s Collaborative. These funders did not have any involvement in study design, data collection, analysis, interpretation, or writing.

## CRediT authorship contribution statement

**Masanori Kurihara:** Writing – original draft, Visualization, Funding acquisition, Formal analysis, Conceptualization. **Ryoko Ihara:** Writing – review & editing, Investigation, Funding acquisition. **Kenichiro Sato:** Writing – review & editing, Validation. **Atsushi Iwata:** Writing – review & editing, Funding acquisition.

## Declaration of competing interest

The authors declare the following financial interests/personal relationships which may be considered as potential competing interests: Masanori Kurihara reports a relationship with 10.13039/501100004896Eisai Inc that includes: speaking and lecture fees. Masanori Kurihara reports a relationship with 10.13039/100019518Eli Lilly that includes: speaking and lecture fees. Ryoko Ihara reports a relationship with 10.13039/501100004896Eisai Inc that includes: speaking and lecture fees. Ryoko Ihara reports a relationship with 10.13039/100014422Eli Lilly that includes: consulting or advisory and speaking and lecture fees. Atsushi Iwata reports a relationship with 10.13039/501100004896Eisai Inc that includes: consulting or advisory, equity or stocks, funding grants, and speaking and lecture fees. Atsushi Iwata reports a relationship with 10.13039/100019518Eli Lilly that includes: consulting or advisory and speaking and lecture fees. Atsushi Iwata reports a relationship with FUJIREBIO Inc that includes: funding grants and speaking and lecture fees. Atsushi Iwata reports a relationship with 10.13039/100017981Sysmex Corporation that includes: funding grants and speaking and lecture fees. Masanori Kurihara has patent pending to FUJIREBIO Inc. Atsushi Iwata has patent pending to FUJIREBIO Inc. KS’s affiliation “Dementia Inclusion and Therapeutics” is an endorsed course funded by Effissimo Capital Management Pte Ltd. If there are other authors, they declare that they have no known competing financial interests or personal relationships that could have appeared to influence the work reported in this paper.

## Appendix Supplementary materials

Image, application 1
