# Supplementary material for: Cost analyses of plasma p-tau217 versus p-tau217/Aβ42 ratio using two-step approach in the Japanese health care system
Source: J Prev Alzheimers Dis. 2026 Apr 18;13(6):100572. doi: 10.1016/j.tjpad.2026.100572 (PMC13098408; doi:10.1016/j.tjpad.2026.100572)
Supplement: Supplementary file 1 [file mmc1.docx]

**Supplementary Table 1.** Breakdown of costs for each estimate

| PET | | | | CSF | | | | |
| --- | --- | --- | --- | --- | --- | --- | --- | --- |
|  | delivery | | tracer synthesis in institution |  | in-patient (one-night admission) | | | lowest out-patient |
|  | flute- metamol | flor- betapir |  |  | our ** estimate | lowest DPC/PDPS | lowest in-patient |  |
| PET-CT procedure | 29,800 | 29,800 | 125,000 | lumbar puncture | 2,700 |  | 2,700 | 2,700 |
| delivery tracer | 196,481 | 184,203 |  | routine CSF | 620 |  | 620 | 620 |
| surcharge | 1,200 | 1,200 | 1,200 | Aβ42/40 (+judgement) | 13,160 |  | 13,160 | 13,160 |
|  |  |  |  | other tests | 5,430 |  |  |  |
|  |  |  |  | admission fee (2 days) | 42,760 | 57,920 | 37,080 |  |
|  |  |  |  | 3 meals | 1,530 | 1,530 | 1,530 |  |
|  |  |  |  | other surcharges† | 62,580 |  |  |  |
| total JPY | 227,481 | 215,203 | 137,000 | total JPY | 128,780 | 59,450 | 55,090 | 16,480 |
| total USD* | 1,550 | 1,460 | 850 | total USD* | 880 | 400 | 370 | 110 |

The lowest cost for amyloid PET in the Japanese health insurance system was 850 USD, when tracers were synthesized within institutions. Although synthesis within institutions may be difficult in the evaluation of AAT where timely on demand testing is necessary (small number of patients per day), we used this cost as the minimal estimate.

The lowest cost estimate for in-patient CSF was 370 USD. Although it could be unrealistic to conduct lumber punctures in hospitals with the lowest admission cost (admission fee + surcharges) where the number of medical staffs are the lowest, we used this cost as the minimal estimate. The lowest cost estimate for out-patient CSF was 110 USD. This was extremely lower than previous estimates in the US (1) or in the United Kingdom (2). Note that one physician and another health care professional (e.g. nurse) are occupied for nearly 30 min. Nevertheless, the procedural cost of lumber puncture is only 2,700 JPY (including local anesthesia) (≈ 18 USD), which is extremely lower than that in the US. Most physicians feel that the price is too low for their limited time and resources in out-patient clinics (3). In our hospital, residents and fellows perform most of the in-patient lumbar punctures.

* approximate equivalent in USD at the currency rate of USD 1 = JPY 147

** in-patient cost estimate in acute-care/teaching hospitals based on that in our institution

† various surcharges based on institutional metrics (e.g. number of nurses per patient)

PET-CT, positron electron tomography-computed tomography; CSF, cerebrospinal fluid; DPC/PDPS, diagnosis procedure combination/per diem payment system; Aβ, amyloid-beta

**Supplementary Table 2.** Sensitivity analyses based on minimal estimated costs for amyloid PET and CSF

|  | PET | | | | in-patient CSF | | | |  | out-patient CSF | |
| --- | --- | --- | --- | --- | --- | --- | --- | --- | --- | --- | --- |
| BBM cost (USD) | delivery flutemetamol | | tracer synthesis in institution | | our estimate | | lowest | | BBM cost (USD) | lowest | |
|  | 1550 USD | | 850 USD | | 880 USD | | 370 USD | |  | 110 USD | |
|  | IMZ 7% | IMZ 14.7% | IMZ 7% | IMZ 14.7% | IMZ 7% | IMZ 14.7% | IMZ 7% | IMZ 14.7% |  | IMZ 7% | IMZ 14.7% |
| 100 | 86.6% | 78.9% | 81.2% | 73.5% | 81.6% | 73.9% | 66.0% | 58.3% | 30 | 65.7% | 58.0% |
| 200 | 80.1% | 72.4% | 69.5% | 61.8% | 70.3% | 62.6% | 39.0% | 31.3% | 40 | 56.6% | 48.9% |
| 300 | 73.7% | 66.0% | 57.7% | 50.0% | 58.9% | 51.2% | 11.9% | 4.2% | 50 | 47.6% | 39.9% |
| 400 | 67.2% | 59.5% | 45.9% | 38.2% | 47.6% | 39.9% | -15.1% | -22.8% | 60 | 38.5% | 30.8% |
| 500 | 60.7% | 53.0% | 34.2% | 26.5% | 36.2% | 28.5% |  |  | 70 | 29.4% | 21.7% |
| 600 | 54.3% | 46.6% | 22.4% | 14.7% | 24.8% | 17.1% |  |  | 80 | 20.3% | 12.6% |
| 700 | 47.8% | 40.1% | 10.7% | 3.0% | 13.5% | 5.8% |  |  | 90 | 11.2% | 3.5% |
| 800 | 41.4% | 33.7% | -1.1% | -8.8% | 2.1% | -5.6% |  |  | 100 | 2.1% | -5.6% |

Sensitivity analyses were conducted by re-calculating cost-saving effect using lowest estimated costs of 850 USD for PET, and 370 (in-patient) and 110 (out-patient) USD for CSF. Cost of BBM was gradually increased until the cost-saving effect (%) turned negative (columns colored in gray).

Cost-saving effect remained positive until 700 USD against PET, 300 USD against in-patient CSF, and 90–100 USD against out-patient CSF.

BBM, blood-based biomarker; PET, positron electron tomography; CSF, cerebrospinal fluid; IMZ, intermediate zone

**Supplementary Table 3.** Sensitivity analyses against PET or CSF based on different intermediate zone percentages

|  | PET | | | | | | CSF | | |
| --- | --- | --- | --- | --- | --- | --- | --- | --- | --- |
| BBM cost per analyte (USD) | delivery flutemetamol | | | tracer synthesis in institution | | | in-patient (one-night) | | |
|  | 1550 USD | | | 850 USD | | | 880 USD | | |
|  | 2 analytes | 1 analyte | differ -ence | 2 analytes | 1 analyte | differ -ence | 2 analytes | 1 analyte | differ -ence |
|  | IMZ 7% | IMZ 14.7% |  | IMZ 7% | IMZ 14.7% |  | IMZ 7% | IMZ 14.7% |  |
| 100 | 80.1% | 78.9% | 1.3% | 69.5% | 73.5% | -4.1% | 70.3% | 73.9% | -3.7% |
| 200 | 67.2% | 72.4% | -5.2% | 45.9% | 61.8% | -15.8% | 47.6% | 62.6% | -15.0% |
| 300 | 54.3% | 66.0% | -11.7% | 22.4% | 50.0% | -27.6% | 24.8% | 51.2% | -26.4% |
| 400 | 41.4% | 59.5% | -18.1% | -1.1% | 38.2% | -39.4% | 2.1% | 39.9% | -37.8% |
| Wang et al.(4) | IMZ 10.7% | IMZ 13% | differ -ence | IMZ 10.7% | IMZ 13% | differ -ence | IMZ 10.7% | IMZ 13% | differ -ence |
| 100 | 76.4% | 80.6% | -4.1% | 65.8% | 75.2% | -9.5% | 66.6% | 75.6% | -9.1% |
| 200 | 63.5% | 74.1% | -10.6% | 42.2% | 63.5% | -21.2% | 43.9% | 64.3% | -20.4% |
| 300 | 49.3% | 67.7% | -18.4% | 18.7% | 51.7% | -33.0% | 21.1% | 52.9% | -31.8% |
| 400 | 37.7% | 61.2% | -23.5% | -4.8 | 39.9% | -44.8% | -1.6% | 41.6% | -43.2% |
| Ishiguro et al.(5) | IMZ 3% | IMZ 15% | differ -ence | IMZ 3% | IMZ 15% | differ -ence | IMZ 3% | IMZ 15% | differ -ence |
| 100 | 84.1% | 78.6% | 5.5% | 73.5% | 73.2% | 0.3% | 74.3% | 73.6% | 0.7% |
| 200 | 71.2% | 72.1% | -0.9% | 49.9% | 61.5% | -11.6% | 51.6% | 62.3% | -10.7% |
| 300 | 58.3% | 65.7% | -7.4% | 26.4% | 49.7% | -23.3% | 28.8% | 50.9% | -22.1% |
| 400 | 45.4% | 59.2% | -13.8% | 2.9% | 37.9% | -35.0% | 6.1% | 39.6% | -33.5% |

Sensitivity analyses were conducted by re-calculating cost-saving effect using different IMZ percentage estimates (1, 4, 5) against PET or CSF. The differences of cost-saving effect (%) are presented with column colored in gray scale when values were negative.

Cost-saving effect remained comparable at 100 USD per analyte and gradually differed as the cost per analyte increased.

BBM, blood-based biomarker; PET, positron electron tomography; CSF, cerebrospinal fluid; IMZ, intermediate zone

**Supplementary Table 4.** Sensitivity analyses against the lowest cost estimates for CSF based on different intermediate zone percentages

|  | in-patient CSF | | | out-patient CSF | | |
| --- | --- | --- | --- | --- | --- | --- |
| BBM cost per analyte (USD) | lowest estimate | | | lowest estimate | | |
|  | 370 USD | | | 110 USD | | |
|  | 2 analytes | 1 analyte | difference | 2 analytes | 1 analyte | difference |
|  | IMZ 7% | IMZ 14.7% |  | IMZ 7% | IMZ 14.7% |  |
| 30 | 76.8% | 77.2% | -0.4% | 38.5% | 58.0% | -19.6% |
| 40 | 71.4% | 74.5% | -3.1% | 20.3% | 48.9% | -28.7% |
| 50 | 66.0% | 71.8% | -5.8% | 2.1% | 39.9% | -37.8% |
| 60 | 60.6% | 69.1% | -8.5% |  | | |
| 70 | 55.2% | 66.4% | -11.2% |  |  |  |
| 80 | 49.8% | 63.7% | -13.9% |  |  |  |
| 90 | 44.4% | 61.0% | -16.6% |  |  |  |
| 100 | 39.0% | 58.3% | -19.3% |  |  |  |
| Wang et al.(4) | IMZ 10.7% | IMZ 13% | difference | IMZ 10.7% | IMZ 13% | difference |
| 30 | 73.1% | 78.9% | -5.8% | 34.8% | 59.7% | -25.0% |
| 40 | 67.7% | 76.2% | -8.5% | 16.6% | 50.6% | -34.1% |
| 50 | 62.3% | 73.5% | -11.2% | -1.6% | 41.6% | -43.2% |
| 60 | 56.9% | 70.8% | -13.9% |  | | |
| 70 | 51.5% | 68.1% | -16.6% |  |  |  |
| 80 | 46.1% | 65.4% | -19.3% |  |  |  |
| 90 | 40.7% | 62.7% | -22.0% |  |  |  |
| 100 | 35.3% | 60.0% | -24.7% |  |  |  |
| Ishiguro et al.(5) | IMZ 3% | IMZ 15% | difference | IMZ 3% | IMZ 15% | difference |
| 30 | 80.8% | 76.9% | 3.9% | 42.5% | 57.7% | -15.2% |
| 40 | 75.4% | 74.2% | 1.2% | 24.3% | 48.6% | -24.3% |
| 50 | 70.0% | 71.5% | -1.5% | 6.1% | 39.6% | -33.5% |
| 60 | 64.6% | 68.8% | -4.2% |  | | |
| 70 | 59.2% | 66.1% | -6.9% |  |  |  |
| 80 | 53.8% | 63.4% | -9.6% |  |  |  |
| 90 | 48.4% | 60.7% | -12.3% |  |  |  |
| 100 | 43.0% | 58.0% | -15.0% |  |  |  |

Sensitivity analyses were conducted by re-calculating cost-saving effects using different IMZ percentage estimates (1, 4, 5) against the lowest estimated costs for CSF in Japan. The differences of cost-saving effect (%) are presented with column colored in gray scale when values were negative. Against the lowest cost estimate of in-patient CSF, cost-saving effect of measuring two analytes (to reduce IMZ) remained comparable in the lowest cost per analyte range. Against out-patient CSF in Japan, cost-saving effect of measuring two analytes was lower than measuring one analyte even in the lowest cost per analyte range.

BBM, blood-based biomarker; PET, positron electron tomography; CSF, cerebrospinal fluid; IMZ, intermediate zone

**References**

1. Palmqvist S, Warmenhoven N, Anastasi F, Pilotto A, Janelidze S, Tideman P, et al. Plasma phospho-tau217 for Alzheimer's disease diagnosis in primary and secondary care using a fully automated platform. Nat Med. 2025;31(6):2036-43.

2. Wittenberg R, Knapp M, Karagiannidou M, Dickson J, Schott J. Economic impacts of introducing diagnostics for mild cognitive impairment Alzheimer's disease patients. Alzheimers Dement (N Y). 2019;5:382-7.

3. Hampel H, Shaw LM, Aisen P, Chen C, Lleo A, Iwatsubo T, et al. State-of-the-art of lumbar puncture and its place in the journey of patients with Alzheimer's disease. Alzheimers Dement. 2022;18(1):159-77.

4. Wang J, Huang S, Lan G, Lai YJ, Wang QH, Chen Y, et al. Diagnostic accuracy of plasma p-tau217/Abeta42 for Alzheimer's disease in clinical and community cohorts. Alzheimers Dement. 2025;21(3):e70038.

5. Ishiguro T, Kurihara M, Nishida Y, Kikkawa-Saito E, Kasuga K, Takenoshita N, et al. Prospective study on clinical utility of plasma p-Tau217 and other biomarkers in Japanese memory clinics using the LUMIPULSE platform. Alzheimers Res Ther. 2026.
